# Diagnosis of gastric mucosal choristoma of the tongue: A case report

**DOI:** 10.1016/j.ijscr.2024.109508

**Published:** 2024-03-11

**Authors:** Saeed Lotfi, Ahmad Madankan, Farzaneh Fazli, Ali Jaliliyan

**Affiliations:** a Department of Internal Medicine, Imam Khomeini Hospital, Tehran University of Medical Sciences, Tehran, Iran; b Department of Surgery, Surgery Research Center, School of Medicine, Rasool-E Akram Hospital, Iran University of Medical Sciences, Tehran, Iran.; c Department of Pathology, Imam Khomeini Hospital, Tehran University of Medical Sciences, Tehran, Iran

**Keywords:** Gastric mucosal choristoma, Ectopic gastric mucosa, Rare tumor, Surgical excision, Histopathology, Glandular structures

## Abstract

**Introduction and importance:**

Gastric mucosal choristoma of the tongue is an extremely rare benign tumor characterized by ectopic gastric mucosa in the tongue. Since first reported in 1927, only around 100 cases have been documented. Herein, we investigated an adult case of Gastric mucosal choristoma who was referred to an ENT clinic with a chief complaint of a solid tumor at the posterior portion of the tongue.

**Case presentation:**

A 32-year-old female presented with a posterior tongue mass initially noticed years ago that progressed over months. A surgical excision was performed. Microscopic examination revealed a gastric mucosal choristoma, with glandular structures resembling gastric mucosa. The postoperative course was uneventful.

**Clinical discussion:**

Lingual gastric choristoma is uncommon but deserves mention due to its rarity. The pathogenesis is unknown but likely represents developmental heterotopia. Clinically, lesions present as asymptomatic tongue nodules often mistaken for more common entities. Thus, histopathology is essential for diagnosis. Microscopy shows gastric mucosa with fundic glands, parietal cells, chief cells, and foveolar epithelium in tongue squamous epithelium.

**Conclusion:**

Gastric choristoma should be considered when evaluating tongue nodules to guide management. Increased awareness of this rare entity can enable accurate diagnosis and treatment. Complete surgical excision is curative with an excellent long-term prognosis. Further study of pathogenesis can elucidate optimal management.

## Highlights

• Rare case of gastric mucosal choristoma in the tongue, presenting as a solid tumor.
• Surgical excision under general anesthesia with meticulous wound closure achieved optimal outcomes.
• Pathological examination confirmed gastric mucosal origin, highlighting distinctive glandular structures.
• Long-term follow-up showed no recurrence or complications, emphasizing successful management.
• Illustrates the importance of accurate diagnosis and management of uncommon lingual masses.

## 1 Introduction

Gastric mucosal choristoma of the tongue is an extremely rare benign tumor characterized by ectopic gastric mucosa in the tongue. Since the first case was reported by Toyama in 1927 [1], only around 100 cases have been documented in the English literature to date. Choristomas are defined as microscopically normal tissue located in an abnormal location and gastric choristoma of the tongue consists of gastric mucosal elements like parietal cells, chief cells, and foveolar cells embedded in the squamous epithelium of the tongue [2].

Clinically, gastric choristoma manifests as a solitary, sessile, painless nodule or polypoid mass ranging from 0.5 to 5 cm in diameter commonly affecting the middle-posterior dorsal surface of the tongue [3]. The lesion is usually soft, smooth, and pink to yellow with occasional surface ulceration. Rarely, multiple lesions may be seen. Due to nonspecific clinical presentation, the differential diagnoses for a tongue lesion would include a wide array of benign and malignant neoplasms like granular cell tumor, neurofibroma, schwannoma, rhabdomyoma, pleomorphic adenoma as well as squamous cell carcinoma [[4], [5], [6]].

Histopathology forms the gold standard for diagnosing gastric choristoma. Hematoxylin and eosin-stained sections show gastric mucosal elements like fundic glands, parietal cells, chief cells, and foveolar epithelium in the squamous epithelium. Gastric foveolar cells may show minimal dysplasia but frank carcinoma is not known to arise from this entity. Immunohistochemistry can help confirm the diagnosis by highlighting gastric epithelial cells positive for mucicarmine, periodic Acid–Schiff, and epithelial membrane antigen stains [3].

Surgical excision is curative and recurrence is infrequent after resection. No malignant transformation has been reported in gastric choristoma. Being a benign lesion, gastric choristoma carries an excellent long-term prognosis after complete excision. Herein, we present a unique case of lingual gastric choristoma in a 32-year-old female patient, successfully treated with surgery alone. The objective of this case report is to increase awareness about this rare entity so that it can be included in the differential diagnosis of a tongue nodule. This can help avoid misdiagnosis and allow timely initiation of appropriate treatment.

## 2 Case presentation

The case report has been reported in line with the SCARE criteria [7]. A 32-year-old female presented with a long-standing history of a solid tumor located at the posterior portion of her tongue. The lesion, initially noticed years ago, underwent a distinctive evolution from an indented structure to a visibly raised mass within a few months before surgical intervention.

Surgical intervention was performed under general anesthesia, involving the complete excision of the lingual mass. The wound was meticulously sutured to ensure optimal healing. Pre-operative CT scan and X-rays had been done for further characterization.

**Fig. 1:**
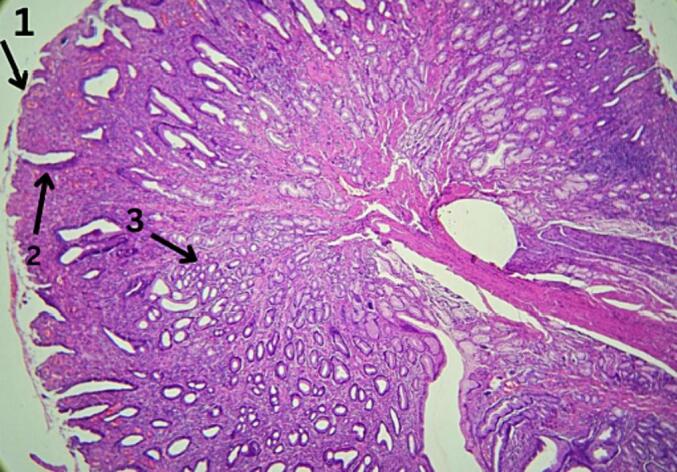
Gastric mucosal choriostoma of tongue.

**Fig. 2:**
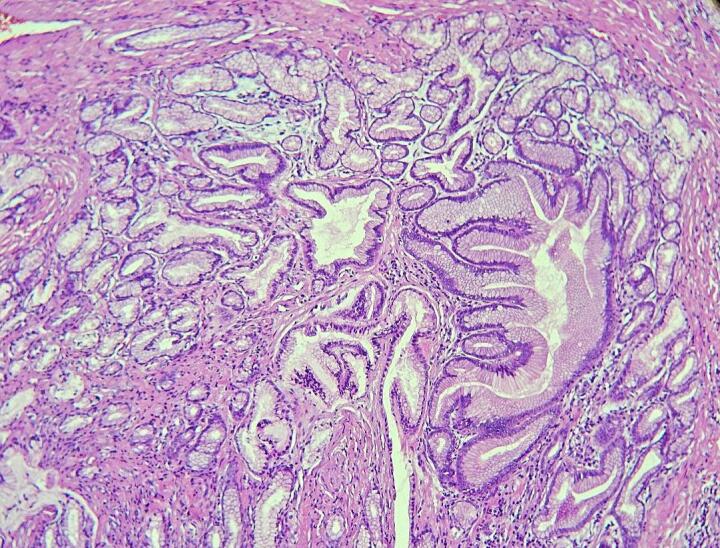
Occupation of back-to-back glands.

Microscopic examination of the excised specimen revealed a gastric mucosal choristoma, a rare histopathological finding within the oral cavity. The pathological investigations illustrated the distinctive features of the choristoma, confirming the gastric mucosal origin, (Fig. 1, Fig. 2). The lesion was partly lined by squamous mucosa (arrow 1), focal erosions were also observable (arrow 2). Also, the deep layer of the specimen was occupied by back-to-back glands (arrow 3). Notably, the lesion exhibited characteristic glandular structures resembling gastric mucosa, validating the diagnosis.

The postoperative course was uneventful, with no signs of recurrence or complications observed during the two-year follow-up period. This case underscores the importance of recognizing and accurately diagnosing uncommon entities such as gastric mucosal choristoma in lingual masses, facilitating appropriate management, and enhancing our understanding of such rare occurrences in clinical practice.

## 3 Discussion

As mentioned, gastric choristoma of the tongue is an uncommon benign lesion that deserves special mention owing to its rarity. The incidence is unknown and likely underreported due to a lack of awareness about this disease entity. Our case adds to the limited knowledge about this rare tumor. What is assumed is that gastric choristoma is mostly observed in children, manifesting clinically at an early age, and is typically addressed through surgical intervention upon patient referral. However, in our case, there hasn't been a significant clinical manifestation until the age of 32, and the patient has not sought medical attention due to it.

This suggests that the growth rate of this tumor varies across different age groups, and it may remain latent until around 30 years of age.

The pathogenesis of lingual gastric choristoma is not well understood. The most plausible theory is that it represents developmental heterotopia of gastric mucosa during foregut embryogenesis. Aberrant differentiation of pluripotent endodermal stem cells results in the sequestration of gastric epithelial components within the squamous epithelium of the tongue. However, trauma, inflammation, or neoplasm have also been implicated as triggers for its development later in life. Additional molecular studies are required to elucidate the exact etiopathogenesis [8].

Clinically, gastric choristomas present as solitary, asymptomatic, sessile nodules on the dorsum of the tongue. Due to nonspecific features, these lesions are often clinically mistaken for more common entities like granular cell tumors, irritation fibroma, lipoma, salivary gland tumors, and even malignancies like squamous cell carcinoma on clinical examination alone. Therefore, histopathology is imperative for accurate diagnosis. The characteristic microscopic finding is the presence of gastric mucosa with fundic glands, parietal cells, chief cells, and foveolar epithelium in the squamous epithelium of the tongue.

Complete surgical excision is the treatment of choice for gastric choristoma. Recurrence is extremely rare after adequate resection. No malignant transformation has been reported indicating its benign biological behavior. Therefore, conservative management with close follow-up may be a reasonable alternative approach for select cases like small lesions or high-risk surgical candidates after ensuring an accurate histological diagnosis. Other treatment modalities like CO2 laser ablation and oral steroid therapy have been rarely described but surgery remains the mainstay of treatment in most reported cases including ours.

In conclusion, gastric choristoma is a rare, benign entity that should be included in the differential diagnosis while evaluating tongue nodules. Increased awareness about this uncommon lesion can help guide appropriate clinical decision-making and management. Histopathology is essential for confirmatory diagnosis. Complete surgical excision is curative and remains the treatment of choice. The long-term prognosis is excellent after adequate treatment. Further studies are required to better understand the pathogenesis which can help determine optimal management approaches.

## 4 Conclusion

In summary, gastric mucosal choristoma of the tongue is an extremely rare benign entity that deserves awareness due to its uncommon nature. Accurate diagnosis requires histopathological examination to identify the characteristic gastric mucosal components. Complete surgical excision remains the treatment of choice with an excellent prognosis. An advanced age at the time of patient referral cannot rule out this diagnosis, and it should be considered in adults as well. Additionally, the growth rate of this tumor may vary over time. If faced with a tumor that has recently increased in size, one may contemplate this diagnosis. Further research into its origins may elucidate optimal management approaches. However, this case highlights that positive outcomes can be achieved with surgery alone for this rare choristoma tumor.

## Patient consent

The patient provided written consent for publication and any associated images.

## Ethical approval

Case report studies are exempt from ethical approval in your institution if no personal information about the patient is included in the article.

### Funding

This research was conducted without any external financial support.

## Author contribution

Lotfi S: Study Concept, Data collection, Data interpretation.

Madankan A: Writing the paper.

Fazli F: Study Concept, Data collection, Data interpretation.

Jaliliyan A: Writing the paper, Data collection.

## Guarantor

Ali Jaliliyan.

## Research registration number

The Case Report is not considered as “First in Man”.

## Conflict of interest statement

The authors declare that they have no conflicts of interest regarding the publication of this research.

## References

[1] Khunamornpong S., Yousukh A., Tananuvat R. (May 1996). Heterotopic gastrointestinal and pancreatic tissue of the tongue. Oral Surg. Oral Med. Oral Pathol. Oral Radiol. Endod..

[2] González Ruiz Y., Cotaina Gracia L., Ruiz De Temiño M., González Esgueda A.J., Delgado Alvira M.R. (2015 Aug). Revisión de casos publicados de coristoma hepático. Diagnóstico diferencial de masas de cordón umbilical. An. Pediatr..

[3] Chang H., Ahn Y., Lim Y.S., Hah J.H. (2009). Gastric choristoma of the oropharynx. Clin. Exp. Otorhinolaryngol..

[4] Zhang Y., Chen M.W.J., Petersson F., Lim A.A.T. (Dec 2023). Solitary plexiform neurofibroma of the tongue: report of a case with no evidence of neurofibromatosis type 1. Int. J. Oral Maxillofac. Surg..

[5] Asadi M., Mohseni M., Jahanshahi F., Esmaeili A., Mohsenifar Z. (Apr 2023). Schwannoma (neurilemmoma) of tongue: a rare case presentation and review of literature. Clin. Case Reports.

[6] Saoud C., McGowan M., Johnson J., Ali S.Z. (Jul 2023). Benign mesenchymal tumours of the tongue: a report of adult-type rhabdomyoma and granular cell tumour with a review of the literature. Cytopathology.

[7] Sohrabi C., Mathew G., Maria N., Kerwan A., Franchi T., Agha R.A. (May 2023). The SCARE 2023 guideline: updating consensus surgical CAse REport (SCARE) guidelines. Int. J. Surg..

[8] Desuter G., Plouin-Gaudon I., De Toeuf C., Gosseye S., Hamoir M. (Jul 2003). Gastric choristoma of the midline neck in a newborn: a case report and review of the literature. J. Pediatr. Surg..

